# Microbiota and short chain fatty acid relationships underlie clinical heterogeneity and identify key microbial targets in irritable bowel syndrome (IBS)

**DOI:** 10.1038/s41598-025-19363-2

**Published:** 2025-10-09

**Authors:** Andrea S. Shin, Yue Xing, Mohammed Rayyan Waseem, Robert Siwiec, Toyia James-Stevenson, Nicholas Rogers, Matthew Bohm, John Wo, Carolyn Lockett, Anita Gupta, Jhalka Kadariya, Evelyn Toh, Rachel Anderson, Amy Dong, Huiping Xu, Xiang Gao

**Affiliations:** 1 Vatche and Tamar Manoukian Division of Digestive Diseases, University of California Los Angeles, Los Angeles, CA USA; 2 Department of Medicine at Stritch School of Medicine, Loyola University Chicago, Chicago, IL USA; 3 Division of Gastroenterology and Hepatology, Indiana University School of Medicine, Indianapolis, IN USA; 4 University of Alabama at Birmingham, Birmingham, AL USA; 5 Department of Microbiology and Immunology, Indiana University School of Medicine, Indianapolis, IN USA; 6 Harvard University, 02138 Cambridge, MA USA; 7 Department of Biostatistics and Health Data Science, Indiana University School of Medicine, Indianapolis, IN USA

**Keywords:** Colonic microflora, Bile acid metabolism, Gut-brain interaction, Functional gastrointestinal disorder, Microbiome, Functional gastrointestinal disorders, Motility disorders, Metagenomics, Microbiome

## Abstract

Short chain fatty acids **(**SCFA) are key microbial metabolites that modulate intestinal homeostasis and may influence irritable bowel syndrome (IBS) pathophysiology. We aimed to assess microbial features associated with SCFA and determine if features varied across IBS subtypes and endophenotypes. We analyzed stool microbial metagenomes, stool SCFA, and measurable IBS traits (stool bile acids, colonic transit, stool form) in 41 patients with IBS (IBS with constipation [IBS-C] IBS with diarrhea [IBS-D]) and 17 healthy controls. We used partial canonical correspondence analyses (pCCA), conditioned on transit, to quantify microbe-SCFA associations across groups. We further compared gut microbiome-encoded potential for substrate utilization across groups and within a subset of participants selected by their stool characteristics as well as stool microbiomes of patients with and without clinical bile acid malabsorption (BAM). Microbe-SCFA associations differed across groups and revealed key taxa including *Dorea* sp. CAG:317 and *Bifidobacterium pseudocatenulatum* in IBS-D and *Akkermansia muciniphila* and *Prevotella copri* in IBS-C that that could underlie subtype-specific microbially-mediated mechanisms. The greatest number of microbe-SCFA associations were observed in IBS-D. Several SCFA-producing species demonstrated inverse correlations with SCFA. Fewer bacterial taxa were associated with acetate to butyrate ratios in IBS compared to health. In participants selected by stool form, we demonstrated differential abundances of microbial genes/pathways for SCFA metabolism and degradation of carbohydrates and mucin across groups. SCFA-producing taxa were reduced in IBS-D patients with BAM. Keystone taxa responsible for SCFA production differ by IBS subtype and traits. IBS microbiomes appear exhibit reduced functional redundancy. Differences in substrate preferences are also linked to bowel functions. Focusing on taxa that drive SCFA profiles and stool form may be a rational strategy for identifying relevant microbial targets in IBS.

**Supplementary Information:**

The online version contains supplementary material available at 10.1038/s41598-025-19363-2.

## Introduction

Irritable bowel syndrome (IBS) is a burdensome disorder of gut-brain interaction with an estimated global prevalence rate of 5–10%^1^. Pathophysiological mechanisms of IBS include disturbances in motility or transit, altered intestinal secretion, impaired intestinal permeability, immune cell reactivity, visceral hypersensitivity, and dysregulated neural signaling and/or central processing^2^. Despite advancements in the understanding of IBS pathogenesis, diagnostic and therapeutic IBS biomarkers are limited.

Accumulating evidence suggests that the gastrointestinal (GI) microbiome is associated with risk of IBS and may also mediate many of the mechanisms that underlie symptoms including altered motility^3,4^, barrier dysfunction^5^, immune activation^6,7^, signaling along the brain-gut axis^8^, and visceral sensation^9^. Characterization of microbial composition in IBS has suggested decreased microbial diversity, reduced temporal stability, or changes in the relative abundance of specific bacteria in patients with IBS^10,11^. However, these findings have not been sufficiently consistent across studies to establish a clear microbial profile in IBS and gaps in our understanding of the functional microbiome persist. Integrating complementary strategies in the investigation of microbial metabolites will be crucial for gathering actionable insights into the impact of the microbiome in IBS.

Atypical profiles of microbial metabolites including luminal bile acids^12,13^ and short chain fatty acids (SCFA) have been described in some patients with IBS^14,15^. Bile acid malabsorption (BAM) is recognized as a mechanistic IBS subtype that can be assessed through several diagnostic methods including measurement of total or primary stool bile acids^13^. Studies have demonstrated BAM to be associated with physiological traits, symptoms, and quality of life^16–19^. Recently, researchers have examined microbial contributions to BAM in IBS to report alterations such as enrichment of *Clostridia* bacteria including *C. scindens*^20^, lower microbial alpha diversity, higher Firmicutes to Bacteroidetes ratio^19^, and presence of endoscopically visible biofilms correlating with overgrowth of *Escherichia coli* and *Ruminococcus gnavus*^21^.

SCFA are produced by anaerobic fermentation of dietary fibers and resistant starch that enter the colon and regulate intestinal homeostasis and physiology^22^. Compared to bile acids, the role of SCFA in IBS is less well understood. Studies of stool SCFA in IBS have yielded variable results, which may be related to the heterogeneity of the IBS patient populations and multiple pathways through which SCFA may modulate intestinal physiology. Therefore, while stool SCFA are unlikely to serve as categorical IBS biomarkers, they may provide critical insights into the pathophysiological mechanisms that underlie IBS symptoms or serve as a tool for identifying metabolically relevant microbial targets. Studies^15,23^ that have assessed stool SCFA in distinct IBS subgroups have reported more consistent associations of stool SCFA with IBS subtypes as well as correlations of stool SCFA with measurable IBS traits such as colonic transit, bowel functions, and bile acid excretion^23–25^. Despite these reports, the intercorrelation between SCFA and transit time complicates the assessment of whether SCFA profiles represent the metabolic capacity of the resident microbiome. It remains unclear if studying the relationships between the microbiome and excreted SCFA is clinically informative. Recent work^26^ has suggested that individual or keystone taxa, rather than complex ecological communities, could drive changes in SCFA output in response to dietary fiber. Therefore, strategies that isolate major microbial features (or keystone taxa) that shape luminal SCFA may serve as a rational method for selecting functionally relevant microbial targets in patients with IBS. To address these questions, we conducted an in-depth investigation of GI microbiome composition and function, stool SCFA, and IBS endophenotypes defined according to quantitative traits (transit, bile acids, bowel functions) in adults with and without IBS.

## Results

### Participant characteristics

**Fig. 1:**
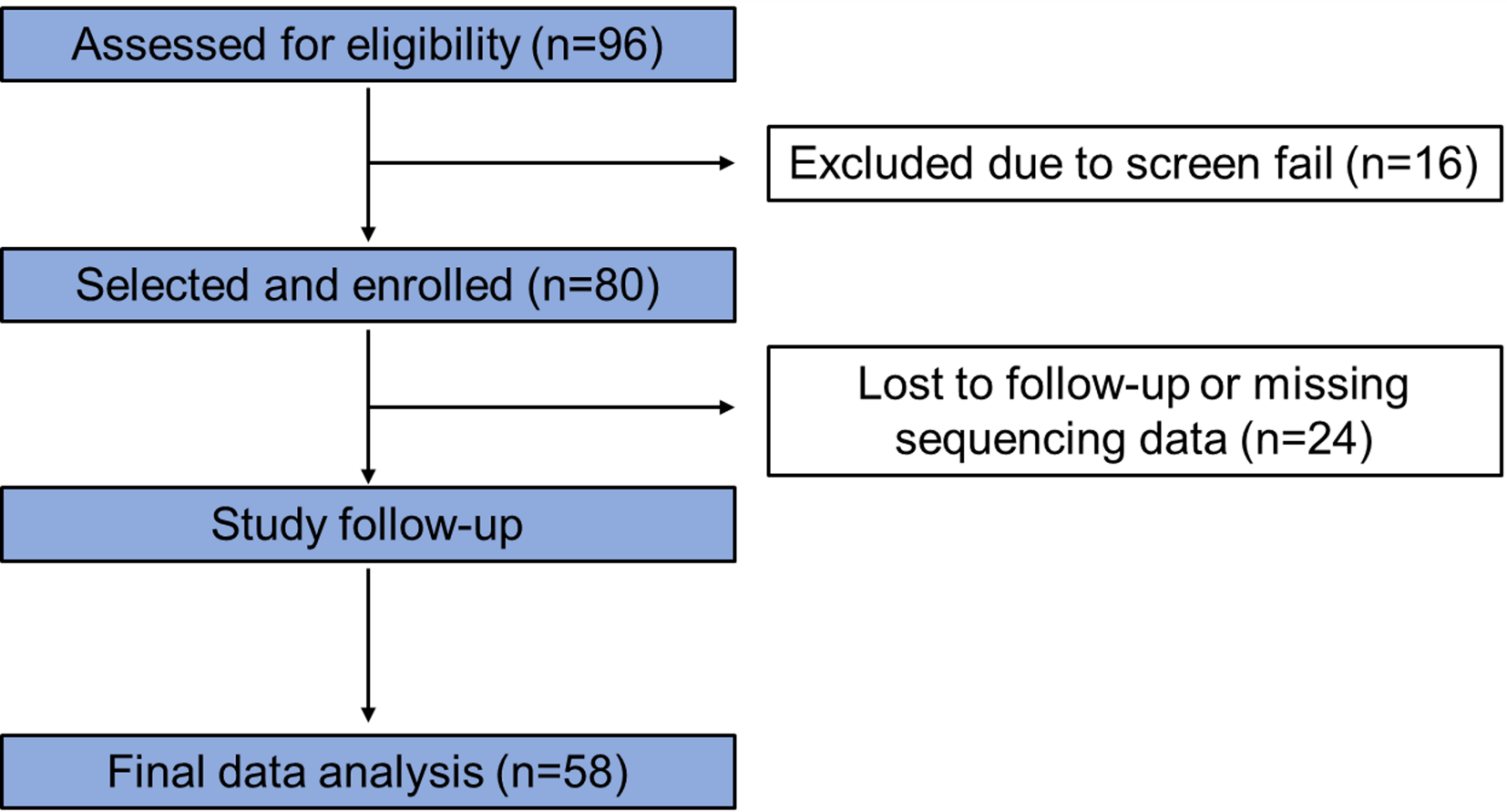
Consort Flow Diagram.

**Fig. 2:**
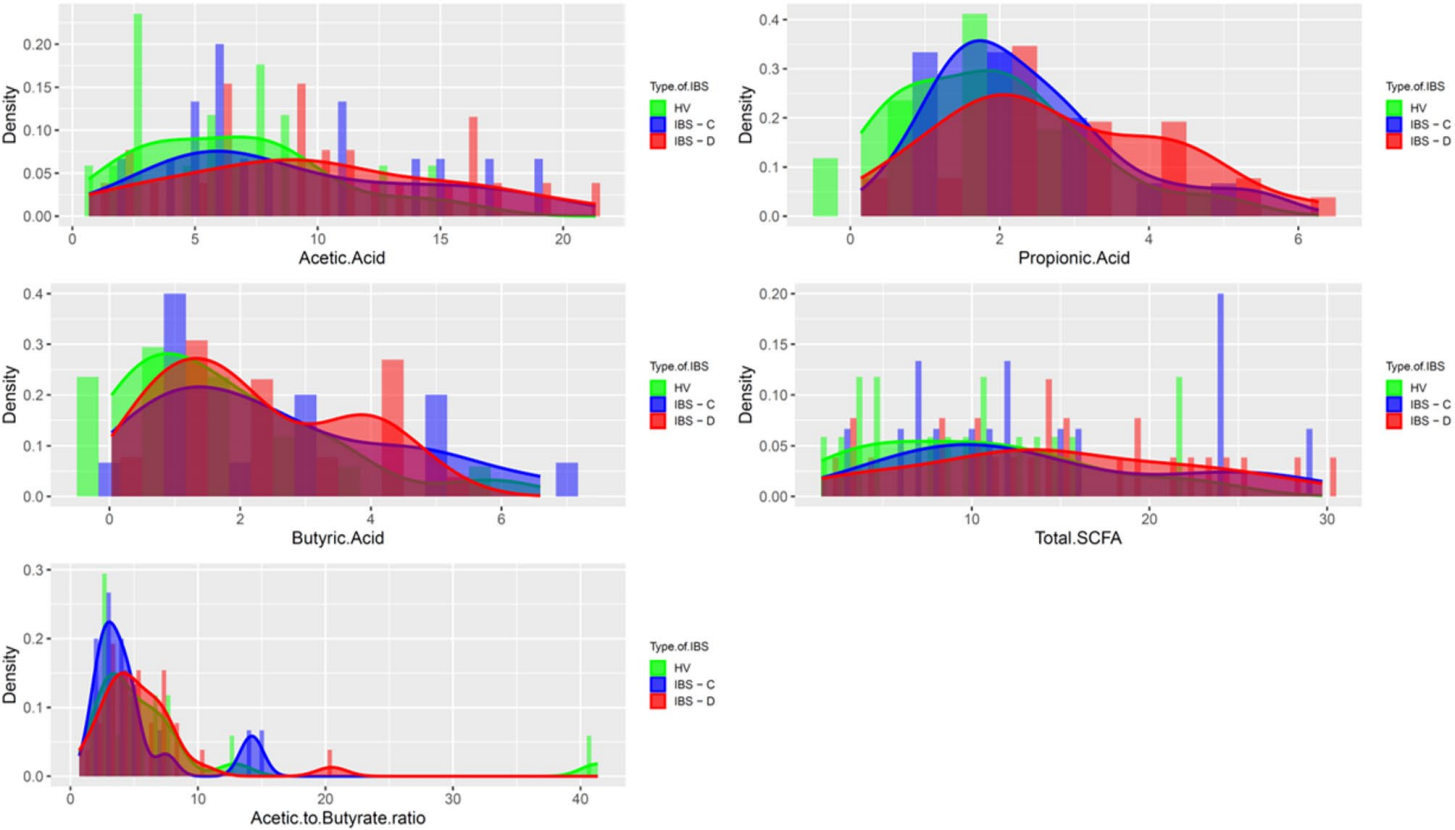
Stool Short Chain Fatty Acid Concentrations in Healthy Volunteers and Participants with Irritable Bowel Syndrome (IBS). *Histograms are shown for total short chain fatty acids*,* acetate*,* butyrate*,* propionate*,* and acetate to butyrate ratios within clinical groups including healthy volunteers (HV)*,* IBS with constipation (IBS-C)*,* and IBS with diarrhea (IBS-D). Groups are denoted by color (HV = green*,* IBS-C = blue*,* IBS-D = red).*

**Fig. 3:**
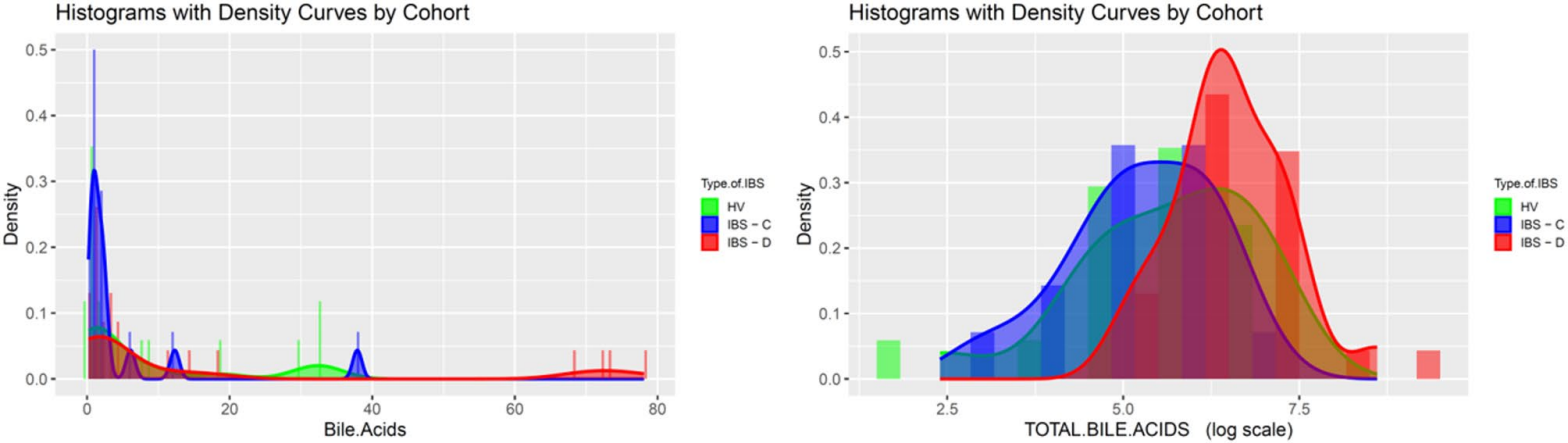
Stool Bile Acids in Healthy Volunteers and Participants with Irritable Bowel Syndrome (IBS). *Histograms are shown for % primary bile acids (bile.acids) and total bile acids within clinical groups including healthy volunteers (HV)*,* IBS with constipation (IBS-C)*,* and IBS with diarrhea (IBS-D). Groups are denoted by color (HV = green*,* IBS-C = blue*,* IBS-D = red).*

Among 96 volunteers who underwent screening evaluation, 71 completed the study, and 58 participants (Fig. 1) with a mean [± SD] age = 35.5 (± 13.8) years and mean [± SD] BMI = 26.2 [± 7.5] kg/m^2^) were included in the final analysis after excluding those who ineligible (*n* = 16), lost to follow-up (*n* = 9), or and/or did not provide SCFA data (*n* = 13). Baseline clinical characteristics (Table 1) including macronutrient intake were not significantly different across groups (all p = ns). No participants reported a historical use of modified, exclusionary, or restrictive diets at the time of screening. Comparisons of quantitative traits demonstrated differences in total stool bile acids and transit between IBS-D and healthy volunteer (HV) participants and in total stool SCFA and stool acetate between IBS and HV Table 2; Figs. 2 and 3.

**Table 1:** Clinical characteristics of patients with irritable bowel syndrome (IBS) and Healthy Volunteers (HV).

Data show mean (standard deviation [SD]) except where specified	HV (*n* = 17)	IBS-C (*n* = 15)	IBS- D (*n* = 26)
**Age (years)**	31.7 (13.6)	34.3 (10.5)	38.7 (15.3)
**Women**,** n (%)**	12 (71%)	13 (87%)	18 (70%)
**BMI**,** kg/m**^**2**^	26.3 (5.9)	25.3 (5.2)	26.7 (9.4)
**White**,** n (%)**	9 (53%)	10 (67%)	23 (88%)
Baseline dietary intake*			
Energy (kcal)	1946.8 (1025.7)	2028.4 (1717.3)	1514.1 (557.4)
Total carbohydrates (g)	219.4 (155.6)	191.1 (153.7)	160.1 (62.9)
Starch (g)	104.7 (54.9)	107.9 (110.9)	87.7 (46.9)
Protein (g)	100.9 (47.1)	96.8 (75.3)	75.1 (23.4)
Fat (g)	77.6 (34.7)	99.6 (101.9)	63.8 (30.3)
Fiber (g)	17.9 (20.9)	14.3 (10.0)	11.6 (4.1)

Group comparisons were conducted using the ANOVA F- and Fisher exact tests. IBS-C, IBS with constipation; IBS-D, IBS with diarrhea; *Diet data missing for two participants with IBS-C and four participants with IBS-D.

**Table 2:** Quantitative traits in patients with irritable bowel syndrome (IBS) and Healthy Volunteers (HV).

Data show median (interquartile range)	HV (*n* = 17)	IBS-C (*n* = 15)	IBS- D (*n* = 26)
Total stool bile acids (µmol/48h)* ^**#$**^	342 (130–640)	190 (110–449)	607 (447–1235)
**Primary stool bile acids (%)**	2.1 (0.8–9.1)	1.8 (0.8–2.4)	3.1 (0.8–12.4)
**Total stool SCFA (µg/mg)** ^@**^	9.8 (4.5–13.8)	11.6 (7.7–20.0)	14.3 (10.0-20.8)
**Stool acetate** ^@*^	6.3 (3.3–8.5)	7.1 (5.3–12.8)	9.4 (5.9–14.1)
**Stool propionate**	1.7 (0.8–2.4)	2.2 (1.5–2.9)	2.4 (1.6-4.0)
**Stool butyrate**	1.2 (0.5–2.4)	2.2 (1.0-3.7)	1.9 (1.0-3.6)
**Acetate to butyrate ratio**	5.0 (3.3–6.7)	3.7 (2.7–4.9)	4.5 (3.3–6.7)
**Total colonic transit time (CTT)**,** days**	1.4 (0.2–2.5)	1.5 (1.0-2.1)	0.9 (0.5–1.5)
**Right CTT**,** days**	0.6 (0.3–0.6)	0.4 (0.2–0.8)	0.4 (0.2–0.6)
**Transverse CTT**,** days**	0.1 (0-0.4)	0.3 (0-0.8)	0.1 (0-0.3)
**Left CTT**, days**^#^	0.5 (0.3–1.9)	0.5 (0.2–1.1)	0.3 (0.1–0.5)

IBS-C = IBS with constipation; IBS-D = IBS with diarrhea; SCFA = short chain fatty acids.

Comparisons across groups were conducted using the Kruskal-Wallis test. For traits showing significant differences (*p < 0.05; **p = 0.05), pairwise comparisons were conducted with Dunn’s test, applying Bonferroni correction for multiple tests. ^#^*p* < 0.05 for HV vs. IBS-D; ^$^*p* = 0.001 for IBS-C vs. IBS-D; all other pairwise comparisons were non-significant (e.g., IBS-C vs. HV); @* *p* < 0.05 for HV vs. IBS. @** *p* = 0.05 for HV vs. IBS.

### Stool microbiome composition differs between IBS and health and between IBS subtypes

Shotgun metagenomic sequencing of stool samples was undertaken to obtain total of 3.1 Gb of sequence data after removal of contaminants with an average of 37.6 million paired-end reads per sample (deposited into NCBI with accession number PRJNA1023929). Taxonomic classification identified 461 taxa at species level classification. Comparisons of microbial metagenomes based on Bray-Curtis Dissimilarity revealed significant divergence (Supplemental Fig. 1) of community distance between groups in both unadjusted analyses (*p* = 0.01) and after adjusting for age, BMI, and dietary variables selected by Random Forest algorithm (*p* = 0.003). We compared abundances of individual taxa across groups using the Kruskal-Wallis test to identify 18 unique and differentially abundant taxa. Highest mean relative abundances of 14 taxa (Supplemental Table 1) including *Dorea* sp. CAG:317, *Blautia* sp. CAG:257, *Ruminococcus gnavus*, and *Proteobacteria* bacterium CAG:139 were observed in IBS-D, which have previously been linked to mechanisms such as BAM^20,21^ and serotonin biosynthesis in patients with IBS-D^27^. *Lawsonibacter asaccharolyticus* abundance was highest in IBS-C and *Firmicutes* bacterium CAG:83 abundance was highest in HV.

**Fig. 4:**
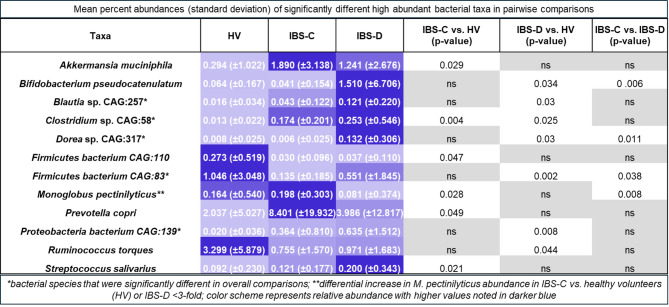
Bacterial Taxa with Differential Abundance of ≥ 3-fold Between Groups. *Data show mean percent abundances (standard deviation) of significantly different bacterial taxa with ≥ 3-fold differences in pairwise comparisons of clinical cohorts.*

We followed unadjusted analyses with pairwise comparisons of ALR-transformed taxa abundances using covariate-adjusted GLM, focusing on high abundant species, to demonstrate significant differences in 17 pairwise comparisons including 12 unique species (Fig. 4) of which 11 exhibited differential abundances of ≥ 3-fold. Among these taxa, we observed significantly higher abundances of *Dorea* sp. CAG:317 and *Bifidobacterium pseudocatenulatum* in IBS-D compared to IBS-C or HV and higher abundances of *Blautia* sp. CAG:257, and *Proteobacteria* bacterium CAG:139 in IBS-D compared to HV. We found significantly higher abundances of *Clostridium* sp. CAG:58 and lower abundances of *Firmicutes* bacterium CAG:83 in both IBS-D and IBS-C relative to HV, respectively. *Akkermansia muciniphila* and *Prevotella copri* were increased in IBS-C compared to HV. Differences of ≥ 3-fold in relative abundances among high abundant bacteria were also observed in 151 pairwise comparisons (Supplemental Table 2) but were not statistically significant after adjustment for covariates.

### Microbe-SCFA associations differ between IBS subtypes and HV

We conducted pCCA on microbiome and SCFA data, conditioned on transit time, to quantify relationships between taxa abundances and stool SCFA in the overall cohort and within clinical groups. In the overall cohort, we observed a significant correlation between microbiome abundance matrix and SCFA concentration matrix (*p* = 0.033, *R*^2^ = 0.095). Relationships between microbiome abundances and SCFA concentrations within clinical groups (Supplemental Fig. 2) were more pronounced within IBS-D (*p* = 0.015) and less pronounced in HV and IBS-C (p = ns). In the overall cohort, the largest positive associations with total SCFA were observed with *Bacteroides plebeius*,* Prevotella sp.* CAG:1031, and *Bifidobacterium pseudocatenulatum*.

### Distinct taxa correlate with individual SCFA across clinical subgroups

**Fig. 5:**
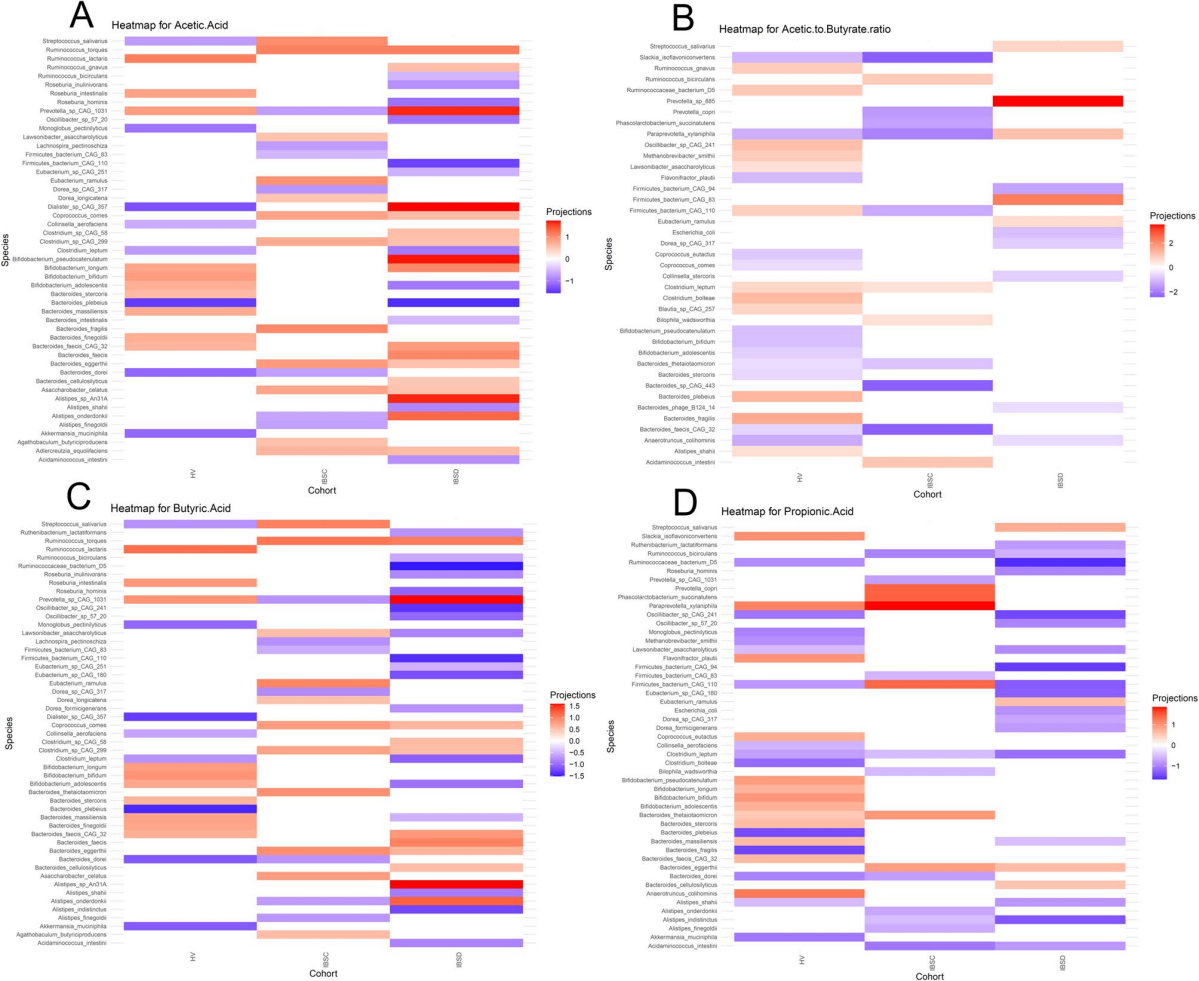
Correlations of bacterial taxa with short chain fatty acids (SCFA) based on partial canonical correspondence analysis. *Panels show associations between bacterial taxa and specific SCFA ([a] acetate, [b] acetate to butyrate ratio, [c] butyrate, and [d] and propionate) within cohorts. The strength of the correlation is represented projections > 0.5 with purple indicating negative correlations and red indicating positive correlations.*

Keeping in mind that SCFA effects may depend on SCFA type, we ranked bacterial taxa according to their projection scores (Supplemental Tables 3–6) for individual SCFAs to assess the pattern of associations between bacterial species and SCFA profiles. A higher projection score for an individual SCFA indicates a greater degree of association with that specific SCFA. Bacteria with projection scores less than 0.5 were excluded from the subsequent analysis. In general, the degree and direction of correlations of individual taxa with acetate, butyrate, and propionate differed according to clinical group (Fig. 5). The greatest number of microbe-SCFA associations were observed in IBS-D, as evidenced by the larger number of bacteria remaining in the ranked list after filtering. Most microbe-SCFA patterns differed between IBS-D and IBS-C and only a few bacterial species including *Ruminococcus torques*, *Coprococcus comes*, *Clostridium* sp. CAG:299, *Bacteroides eggerthii*, and *Adlercreutzia equolifaciens* demonstrated consistent associations across both IBS subtypes with acetate and butyrate.

In IBS-D, both positive and negative correlations with stool acetate were observed with multiple taxa. Largest negative projections were observed with several bacteria that have previously linked to SCFA production (*B. plebeius*, *Roseburia hominis*)^28,29^, dietary polysaccharides and protein utilizers (*Firmicutes bacterium* CAG:110)^30^, starch or fructooligosaccharide degradation (*Bifidobacterium adolescentis*), fiber fermentation (e.g. *Clostridium leptum*^31^, β-fructan utilizers (e.g. *Roseburia inulinivorans*)^31^. Positive projections were observed with *Dialister* sp. CAG 357, *B. pseudocatenulatum*, *Alistipes* species, *Prevotella* sp. CAG:1031, *R. toques*, and *R. gnavus.* In IBS-C, largest negative projections for acetate were observed between abundances of *Dorea* sp. CAG:317, *Lachnospira pectinoschiza*, and *Prevotella sp.* CAG:1031 while the largest positive projections were observed with *Streptococcus salivarus*, *Bacteroides fragilis*, and *R. torques*. In HV, largest negative projections for acetate were observed with *B. plebeius*,* Dialister* sp. CAG:357, *Bacteroides dorei*, and *A. muciniphila*. The largest positive projection for acetate among HV was observed with *Ruminococcus lactaris*.

Analysis of microbe-butyrate associations in IBS-D demonstrated negative projection scores for butyrate with several bacteria including *Ruminococcaceae* bacterium D5, *Firmicutes bacterium* CAG:110, *C. leptum*, *R. hominis*,* R. inulinivorans*, and *L. asaccharolyticus*. The largest positive projection scores for butyrate were observed with *Alistipes* species, *Prevotella sp.* CAG:1031, and *R. torques*. In IBS-C and HV, patterns of microbe-butyrate associations largely mirrored the patterns of microbe-acetate associations observed within the respective clinical groups. In addition, a positive relationship between butyrate and *Bacteroides thetaiotaomicron*, a well-known human commensal^32,33^ that exhibits the capacity to digest a broad array of polysaccharides and host glycans, was also observed in IBS-C.

For propionate, most microbes including *Ruminococcaceae* bacterium D5, *Firmicutes bacterium* CAG:110, *C. leptum*, *L. asaccharolyticus*, *Ruthenibacterium lactatiformans* and *Bacteroides massiliensis* exhibited negative associations in IBS-D with only a few exceptions including *S. salivarus*, for which the largest positive correlation was observed. In contrast, both positive and negative projections for stool propionate were observed in IBS-C; the largest positive scores were observed for *Paraprevotella xylaniphila*, *Phascolarctobacterium succinatutens*, and *P. copri*. Among HV, both positive and negative associations with propionate were observed for multiple bacterial taxa.

### Network-based validation of microbe–metabolite associations

The results of Bayesian network analyses (Supplemental Fig. 3) revealed consistent patterns with the projection score analysis. Notably, many taxa with high projection scores for individual SCFAs (e.g., *R. torques*, *R. hominis*,* R. inulinivorans*, *C. leptum*, *B. plebeius*, *Prevotella sp. CAG:1031*, etc.) were also found to be directly or indirectly associated with SCFAs in the Bayesian networks.

### Lower number of bacterial species associated with acetate to butyrate ratios in IBS

Since interconversion of SCFA from acetate to butyrate may represent a major route of butyrate formation within the gastrointestinal tract^34^, we examined bacterial relationships with acetate to butyrate ratios to identify top taxa associated the metabolic processes that drive SCFA output. The largest positive associations were observed with *Prevotell*a sp. 885, *Firmicutes* bacterium CAG:83, *P. xylaniphila* in IBS-D, suggesting that these species were associated with shift toward decreased butyrate production. Unlike the patterns observed with individual SCFA, analysis acetate to butyrate ratios demonstrated that the number of ranked bacteria were substantially lower in IBS-D or IBS-C compared to controls (Fig. 5), suggesting the possibility of reduced functional redundancy for butyrate formation in IBS.

### Reduced SCFA-producing microbes and bile acid malabsorption (BAM)

Stool microbial metagenomes were analyzed in 22 IBS-D patients with (*n* = 8) and without (*n* = 14) clinical BAM according to previously validated diagnostic cutoff values^12,13^. Comparisons of beta diversity based on Euclidian dissimilarity of ALR transformed abundance data showed significant dissimilarity (*p* = 0.039; R^2^ = 0.061) between patients with and without BAM (Supplemental Fig. 4). Significant differences in six high abundant species were observed between groups (Supplemental Table 7), including *L. asaccharolyticus R. inulinivorans*, *L. pectinoschiza*, and *F. saccharivorans* which were also associated with stool SCFA, but not bile acids, in IBS-D. These four species with known SCFA-producing capacity were negatively correlated with BAM.

### Functional potential for carbohydrate degradation, SCFA metabolism, and mucin degradation

**Fig. 6:**
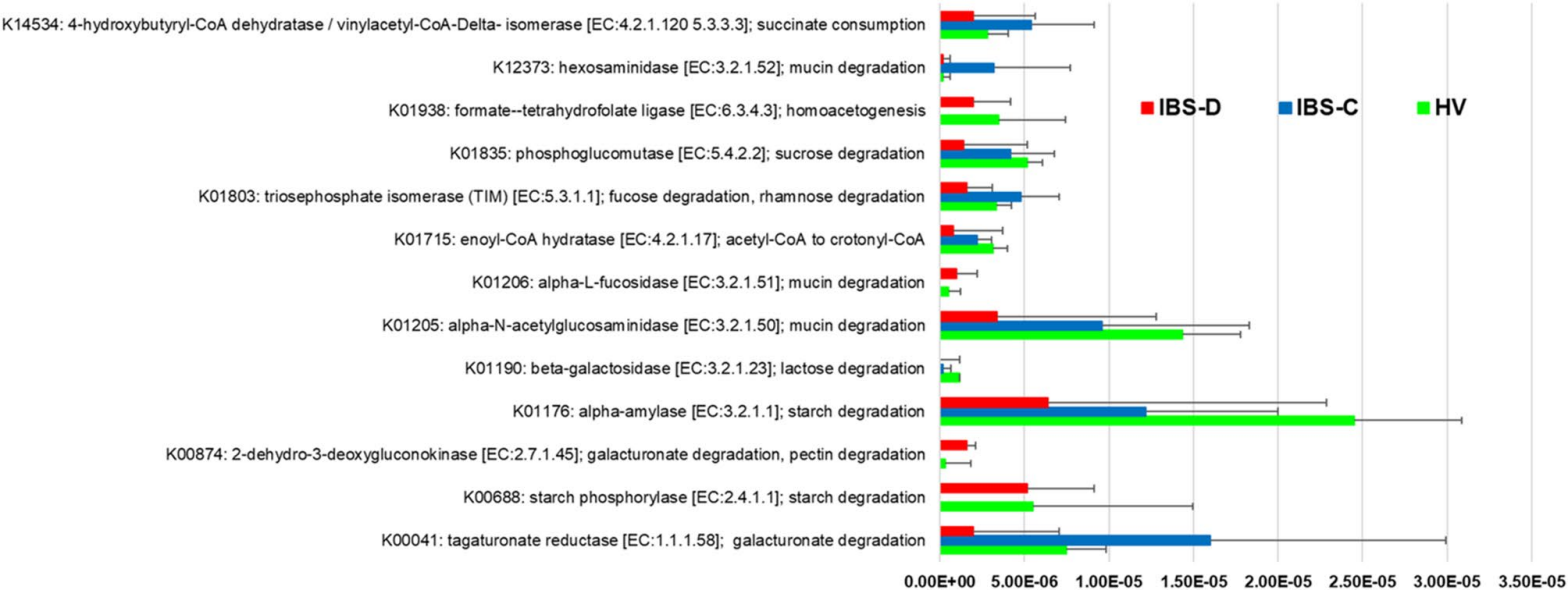
Variations in Microbial Substrate Utilization in Healthy Volunteers and Participants with IBS. *Figure shows significant differences in functional potential for carbohydrate degradation*,* short chain fatty acid production or metabolism*,* and mucin degradation across clinical groups in representative subset selected by stool form characteristics. Groups are denoted by color (HV = blue*,* IBS-C = red*,* IBS-D = gray). *. *Permission through Kanehisa Laboratories was not requested as* Fig. 6*was independently generated and we did not use any images or diagrams from Kanehisa Laboratories.*

Examination of microbial gene families and pathways yielded 949,223 total gene families within the metagenome data set including 4,042 named KO terms. We applied a gut metabolic module (GMM) framework to analyze modules related to carbohydrate degradation, SCFA production or metabolism including bacterial cross-feeding pathways, mucin degradation, and one module related to serine degradation based on significant species-based predictors. Abundances of KO identifiers within modules for lactose degradation, (*p* = 0.0026), serine degradation (*p* = 0.019), and propionate production (*p* = 0.026) were significantly enriched in IBS-C compared to controls (Supplemental Table 8). Relative abundance of a KO identifier within a galactose degradation module was significantly reduced in IBS-D (*p* = 0.033) compared to controls. In the collective cohort, we applied the GMM framework to analyze the relationships between metagenomically-encoded functions and stool SCFA. We identified 23, 23, 15, and 16 KO identifiers from relevant GMMs that were significantly associated with total SCFA, acetate, butyrate, and propionate, respectively (Supplemental Tables 9–12). Within a representative subset (*n* = 6 controls, *n* = 5 IBS-D, *n* = 5 IBS-C) selected by stool form characteristics, functional metagenomic analyses demonstrated that microbial genes/pathways associated with SCFA production/metabolism and degradation of carbohydrates/mucin were differentially associated with clinical group (Fig. 6).

## Discussion

It has been proposed that changes in intestinal SCFAs in some patients with IBS are caused by shifts in microbial composition and function to drive and maintain symptoms. We hypothesized that specific microbiome features correlate with SCFA output and that these relationships vary across IBS subtypes and endophenotypes. Using a dual-omics approach, we found that microbiota-SCFA relationships differ between IBS subtypes and endophenotypes, and between patients with IBS and HV. The focus on paired microbe-SCFA data was grounded in the known biological relevance of SCFA in GI physiology. Our findings suggest that key taxa may shape the microbial metabolome, contribute to IBS mechanisms, and influence bowel functions through their functional capacities.

While many studies have reported compositional changes^11^ in IBS, consistent patterns are elusive. In our study, we observed differences in overall microbial community composition across groups with significant changes being most apparent in IBS-D. These alterations were predominantly characterized by higher abundances of several bacterial species including *Blautia* sp. CAG:257, *R. gnavus*, *Dorea* sp. CAG:317, and *Proteobacteria* bacterium CAG:139. *R. gnavus*, has previously been implicated in IBS-D pathogenesis through serotonin biosynthesis^27^, biofilm formation and increased stool bile acid excretion^21^, production of proinflammatory polysaccharides^35^, and mucin degradation^36^. Interestingly, the relationship between *R. gnavus* and IBS lost its significant in adjusted analyses due to a positive association between *R. gnavus* and BMI, which may suggest a role for *R. gnavus* in explaining the connection between IBS-D or chronic diarrhea with metabolic syndrome and obesity-related disorders^37^. Associations of IBS-D with other taxa including *Dorea* sp. CAG:317, *Blautia* sp. CAG:257, and *Proteobacteria* bacterium CAG:139 remained and a new association between *B. pseudocatenulatum* and IBS-D emerged.

In our analyses of microbe-SCFA relationships, largest contributions to SCFA in the overall cohort were observed with *B. plebeius*,* Prevotella sp.* CAG:1031, and *B. pseudocatenulatum*, indicating their central role in microbial metabolism. These taxa possess adaptations for dietary carbohydrate fermentation, supporting their metabolic relevance^38–41^. In IBS-D, microbe-SCFA associations were most pronounced in IBS-D and fewer microbes were linked to acetate and butyrate ratios. These observations may indicate that microbial contributions to SCFA profiles are particularly important in IBS-D and could also imply a greater degree of microbial specialization and reduced functional redundancy. Previously, Jacobs et al.^42^ reported significant increases in transcript abundances for fructooligosaccharide and polyol utilization and upregulation of transcripts for fructose and glucan metabolism as well as the succinate pathway for carbohydrate fermentation in IBS. Comparisons between IBS-D and IBS-C demonstrated differences in microbial metabolism with upregulation of multiple metabolic pathways including transcripts for fructose, mannose, and polyol metabolism in IBS-D. Together, our findings suggest that differences microbial metabolism may underlie variations in clinical symptoms or pathophysiological mechanisms across IBS subtypes and that altered microbial metabolism could be particularly important in IBS-D. Our findings may further explain the higher prevalence of mixed- and diarrhea-predominant symptoms in post-infection IBS^43,44^ as well as the limited evidence for efficacy of antimicrobial treatments such as rifaximin in IBS-C^45^ relative to IBS-D^46,47^.

*Dorea* sp. CAG:317 and *B. pseudocatenulatum* were identified as top ranked bacteria associated with acetate to butyrate ratio and acetate concentrations. Mucin degradation has been described among several *Dorea* species and the genus *Dorea* belongs to the Lachnospiraceae family, a major producer of SCFA^48^, Notably, Wang et al.^49^ recently demonstrated that while xylan supplementation following fiber deprivation alleviated gut dysbiosis by promoting *B. pseudocatenulatum*, these changes were associated with lower community diversity. Therefore, *Dorea* sp. CAG:317 and *B. pseudocatenulatum* may represent keystone taxa linked to altered microbial metabolism or persistent impairment in community recovery among patients with IBS-D. Only two bacterial species, *A. muciniphila* and *P. copri* were significantly increased in IBS-C. *P. copri* was further identified as top ranked bacteria that was positively associated with propionate and negatively associated with acetate to butyrate ratio in IBS-C. Previous studies have reported a positive association between mucosal *P. copri* abundance and abdominal pain in patients with IBS without prior history of infection^50^. Others have demonstrated that IBS patients with high levels of propionate present with worse gastrointestinal symptoms, quality of life, and negative emotions^15^. *P. copri* is a prominent gut commensal that contributes to propionate production via the succinate pathway^51^ and has been linked to both beneficial and harmful effects on human health. In another study by Jiang et al., *P. copri* colonization with high fiber diets was associated with proinflammatory effects and *Akkermansia* expansion in patients with rheumatoid arthritis^52^. Together, these observations may indicate *P. copri*–driven propionate production, particularly in the context of increased *Akkermansia* could promote pathophysiological mechanisms (e.g., immune activation) that drive symptom burden in in IBS-C.

In addition to identify major microbial features associated with individual SCFA, we made several important observations related to the pattern or direction of observed microbe-SCFA relationships. Interestingly, negative correlations were observed in the IBS-D subgroup between several SCFA-producing species including *R. hominis*,* B. adolescentis*, *R. inulinivorans* and individual SCFA. Findings may imply disrupted cross-feeding mechanisms, modifications in substrate utilization, competitive inhibition, or other context-specific changes leading to altered microbial metabolism in IBS. *R. inulinivorans* is a known fiber fermenter^31^ that also possesses the ability to forage mucin through a metabolic interplay with other members of the intestinal microbiota^53^ and is known for net acetate uptake^51^, which may explain its correlation with reduced SCFA. Upon analyzing relationships of bacterial species with acetate to butyrate ratios, which we studied as a marker for overall SCFA production and luminal SCFA availability^54^, we identified fewer top ranked bacteria in IBS compared to healthy controls, suggesting a reduction in overall functional redundancy of the IBS microbiome. Future work should investigate the effects of bacterial cross-feeding, microbial conversions, and interactions of the colonic microbiome with diet- and host-derived substrates that may destabilize the microbiota-mucosal interface to expose vulnerabilities and that drive IBS pathogenesis.

A few bacterial species were associated with SCFA in both IBS subtypes including *R. torques*, *C. comes*, *Clostridium* sp. CAG:299, *B. eggerthii*, and *Adlercreutzia equolifaciens* which may suggest these bacterial species represent shared microbial features across IBS subtypes. *R. torques* is a mucin-degrading bacteria that has previously been associated with IBS^55^ as well as depression^56^. Similarly, recent studies have demonstrated associations between *C. comes*^57^ and *B. eggerthii*^58^ depression and post-traumatic stress disorder, which have both been described as comorbid conditions in IBS. These data could suggest that some microbial features are linked to common pathophysiologic mechanisms involving the microbiota-gut-brain axis that are shared across IBS subtypes and that their effects may be mediated by microbially-derived SCFA.

In our comparisons across IBS-D patients by with and without BAM, we observed BAM to be associated with reduced abundances of several SCFA-producing species. Similar observations have been made in studies linking biofilm formation to bile acid accumulation and reduced SCFA-producing bacteria^21^. Results suggest that BAM exerts indirect effects on the intestinal metabolome and may associated with a decline in SCFA-producing capacity. We also assessed of functional variations in the GI microbiome to find differential abundances of KO identifiers for metabolic processes related to degradation of carbohydrates and serine as well as SCFA production. In a subset of participants selected by stool form characteristics, we found differences in substrate degradation potential and fermentative capacity across groups including increased capacity for starch degradation in controls, alterations in distinct mucin-degrading functions in IBS-D vs. IBS-C, and reduced pectin degrading potential in IBS-C. Results suggest that abnormal stool form in IBS is influenced by microbiota-encoded substrate preferences.

Collectively, our findings indicate there are distinct changes in microbiome composition may underlie clinical heterogeneity in IBS. We demonstrate that the gut microbiota in IBS-D is characterized by increased metabolic activity, reduced functional redundancy, and persistently reduced diversity. Several taxa including *R. gnavus*, *Dorea* sp. CAG:317 and *B. pseudocatenulatum* emerged as the leading candidates for further study as potential microbial targets in IBS-D. A few unique microbial features including *P. copri*-associated propionate production and *Akkermansia* expansion may play a crucial role in IBS-C. Meanwhile, microbial features that are common between subtypes may offer insights into shared pathophysiological mechanisms involving the microbiota-gut-brain axis in IBS. Identifying the major microbial features (taxa or functions) that drive metabolic output may be crucial in defining rational approaches to modulate the microbiome in IBS.

Despite study strengths, we recognize the limitations of this work including cross-sectional sampling, which overlooks the fluctuating nature of the microbiome. To address this, we assessed relationships between microbial abundances and quantifiable IBS endophenotypes that were defined at the time of specimen collection while accounting for baseline covariates. All participants were instructed to consume a 4-day high fat diet while otherwise maintaining their usual diet. Previously, we examined the effect of both habitual diet and real-time intake on excreted stool SCFA to find that that modest variations in macronutrient intake including polysaccharides did not exert substantial effects on excreted SCFA^25,59^. We enrolled patients with opposing IBS phenotypes from both the university clinics and the surrounding communities to increase our ability to detect differences between clinical cohorts and limit referral bias. We also acknowledge that causal microbial mechanisms cannot be determined through the current work. However, we applied a dual-omics approach to investigate biologically relevant changes in intestinal microbiome composition and function. We cannot discount the possibility we may have missed significant taxa due to the moderate sample size and a risk for Type I errors as we did not adjust for false discovery rate in our analyses of taxonomic differences. Moreover, the choice of metagenomic classification tool may influence taxonomic profiling^60^. Hence, results should be considered as hypothesis-generating. However, our analytic approach was based on endpoints that were determined a priori and we applied a conservative strategy by focusing on taxa that were associated with SCFA while accounting for transit. We further limited analyses to high abundant taxa and to species that exhibited > 3-fold change in abundance while focusing on the biological plausibility of our results. MetaPhlAn is a widely used metagenomic classification tool that relies on clade-specific marker genes is well-suited for identifying species-level patterns in human samples. A complementary Bayesian Network Analysis was also conducted to better capture microbial interactions and shared influences on SCFA profiles. Importantly, many of the taxa identified via projection scores were noted to occupy structurally important positions in the broader microbiome-metabolite-transit interaction network, supporting the biological relevance of our findings. Finally, we compared IBS subtypes, but did not evaluate symptom severity with tools such as the IBS severity scoring system^61^, which others have shown may be correlated with microbiome composition^3^. Findings from this study should be validated in larger, longitudinal cohorts and investigate other aspects of IBS phenotypes such as symptom severity, but still provide a novel framework that could be used to uncover key microbial interactions and targets in IBS.

## Conclusions

In conclusion, main findings from this study highlight microbiota-SCFA patterns vary across clinical IBS phenotypes and endophenotypes. Prominent shifts in microbial composition are observed in IBS-D. These changes appear to affect metabolic capacity, substrate preferences, and metabolite profiles. In addition, altered microbial substrate uptake may impact stool form or bowel function in IBS. Importantly, we identify specific taxa—such as *R. gnavus*, *Dorea* spp., and *B. pseudocatenulatum*—that may serve as biomarkers or therapeutic targets due to their strong association with SCFA profiles. Our findings support the utility of a paired microbe-metabolite approach as a viable strategy for identifying functionally relevant microbial features in heterogenous IBS patient populations.

## Materials and methods

### Participant recruitment and study design

The study was approved by the Indiana University Institutional Review Board and the protocol registered within ClinicalTrials.gov (NCT02981888). The study was conducted in accordance with the principles of the Declaration of Helsinki and all patients provided written informed consent before enrollment. The study was designed as an observational investigation of stool SCFA, stool bile acids, colonic transit, and stool microbiota in adults with and without IBS. We enrolled adults ages 18–65 years of age through the Indiana University Gastroenterology Clinics, the Indiana Clinical and Translational Research Institute Research Registry, and from the local community. We included individuals with IBS with diarrhea (IBS-D) or IBS with constipation (IBS-C) according to Rome IV criteria^62^ and healthy controls with no prior history of GI diseases or symptoms. Detailed eligibility criteria are available in the Supplemental Methods.

### Data collection

Study eligibility, medications, medical history, and baseline diet using a food frequency questionnaire^63^ were assessed during a screening visit with a study physician. Over a two-week period, data were collected on bowel functions using a standardized bowel pattern diary including the Bristol stool form scale^64^. All participants were instructed to maintain their habitual diets and submitted a 48-hour stool collection collected during the last 2 days of a 4-day 100 g fat diet, consistent with clinically validated methods for identifying BAM. Specimens were refrigerated during the collection period, returned to the research team on ice, and stored at −80^ο^C.

### Colonic transit by radiopaque markers

Participants underwent assessment of colonic transit time with a previously validated and optimized method using radiopaque markers^65^.

### Stool SCFA and bile acids

Frozen aliquots of stool were shipped to the Metabolite Profiling Facility at Purdue University to measure total and individual SCFA concentrations per mg of dry weight by liquid chromatography-mass spectrometry (LC-MS) using published methods^66^ and to the Mayo Clinic Department of Laboratory Medicine and Pathology to measure total and primary stool bile acid levels by high-performance LC-MS through a commercially available, CLIA-approved assay^16,67,68^. Individual SCFA of interest included acetate, butyrate, and propionate as these represent the predominant SCFA produced in humans^69^.

### DNA extraction, purification, and sequencing

Genomic DNA was isolated from stool using the QIAmp^®^ PowerFecal^®^ DNA kit (QIAGEN Inc., Germantown, MD, USA). DNA quality and concentration were on a Qubit fluorometer. Purified DNA underwent library preparation (Nextera XT, Illumina) and paired-end (2 × 150 bp) sequencing using the NovaSeq v1.5 SP (Illumina, San Diego, CA, USA) to target a sequencing depth of 40 M sequences per sample.

### Metagenomic data analysis

Metagenomic sequencing reads were quality filtered and processed for taxonomic profiling using MetaPhlAn3. Additive log-ratio (ALR) transformation was applied to analyze differential taxonomic abundances. Based on the identified microbial taxa from each sample, β-diversity indices (e.g., Bray-Curtis dissimilarity) were calculated using the R packages phyloseq and vegan^70,71^. Functional profiling was conducted using the HUMAnN3 pipeline annotated by KEGG (Kyoto Encyclopedia of Genes and Genomes) orthogroups (KO)^72–74^.

### Statistical considerations

We summarized major endpoints of interest: (1) stool microbiome composition, (2) stool SCFA concentrations (total, acetate, propionate, butyrate), total and percent primary stool bile acids, and (3) colonic transit time (overall and segmental). Our primary objective was to quantify the association between microbiome composition and SCFA (microbe-SCFA associations) across clinical groups after controlling for transit and to account for the mechanistic heterogeneity that underlies clinical IBS populations.

For overall community composition, we compared microbial community dissimilarity using the PERMANOVA test across groups (IBS-C, IBS-D, controls) with and without adjusting for covariates (age, BMI, dietary variables) selected by the Random Forest algorithm. Dietary variables suggested by the machine-learning technique included starch, total energy, and protein. Significant associations of community distance by Bray-Curtis Dissimilarity with group were observed in both diet-adjusted and unadjusted analyses. We compared microbial taxa abundances between groups and analyzed associations of taxa abundances with IBS endophenotypes/traits (stool SCFA, stool bile acids, and transit) for the collective cohort and within groups. Associations of microbiome composition with group were assessed using general linear regression models (GLM) adjusted for covariates including age, sex, and BMI. Only high abundant species (relative abundance ≥ 0.1%) that were prevalent in ≥ 2 specimens within ≥ 1 clinical group were considered. Partial Canonical Correspondence Analysis (pCCA) conditioned on transit time was employed to Quantify the relationship between the microbiome and stool SCFA in all participants and within clinical groups, using the vegan package in R. Conditioning on transit time accounted for variation in microbial and metabolite profiles attributable solely to differences in gastrointestinal transit. The significance of the model was assessed through a permutation test with 999 iterations. To identify bacteria whose abundances exhibited the greatest degree of association with the stool SCFA concentrations, we focused on those bacteria scattered along the direction of specific SCFA axis in the biplot, within a 60-degree angle centered around the SCFA axis in both positive and negative directions. The strength of association between the bacteria and the SCFA was ranked by projecting of the bacteria species scores onto each SCFA axis in the biplot. Bacteria with projection scores less than 0.5 were excluded from the subsequent analysis. To complement the projection score approach and explore the complexity of microbiome–metabolite–transit interactions, we conducted a Bayesian Network Analysis with R package bnlearn. The network structure was inferred using a bootstrapped ensemble of 500 iterations and only edges that appeared in at least 50% of bootstraps were retained. All associations were included (i.e., partial correlation coefficient > 0).

For exploratory analyses, we analyzed multivariable associations of microbiome composition with clinical BAM among individuals with IBS-D. Relative abundances were used to examine the associations of gene family/pathway abundances across clinical groups. A manually curated gut-metabolic analysis framework^75^ was applied to examine KO identifiers associated with carbohydrate degradation, SCFA production or metabolism, and mucin degradation using GLM adjusting for covariates. As stool form^76^ has been identified as a strong source of human microbiota variation and closely linked to transit^77,78^, we further explored associations of functional potential across groups in a subset of participants who were selected based on stool form features (i.e. those individuals best representing the stool types within their respective clinical group according to bowel diary data) using the Kruskal-Wallis test. For example, we chose the IBS-D and IBS-C participants with the loosest and firmest stool types, respectively, and controls with consistently normal stool types. For all endpoints, missing values were excluded from the analysis for that endpoint.

## Supplementary Information

Below is the link to the electronic supplementary material.

Supplementary Material 1

Supplementary Material 2

Supplementary Material 3

Supplementary Material 4

Supplementary Material 5

Supplementary Material 6

## Data Availability

Microbiome sequencing reads are deposited into NCBI with accession number PRJNA1023929. All relevant data generated during the study and the R codes used to conduct the analyses for this study are included in this published article (and its supplementary information files) or are available for download from GitHub via the following link: https://github.com/xianggao2006/IBS (accessed on 1 May 2025).

## Abbreviations

ALR: Additive log-ratio
BAM: Bile acid malabsorption
CTT: Colonic transit time
gDNA: Genomic DNA
GI: Gastrointestinal
GLM: Generalized linear model
GMM: Gut metabolic module
HV: Healthy volunteer
IBS: Irritable bowel syndrome
IBS-D: IBS with diarrhea
IBS-C: IBS with constipation
KEGG: Kyoto Encyclopedia of Genes and Genomics
KO: KEGG Orthogroup
LC-MS: Liquid chromatography-mass spectrometry
SCFA: Short chain fatty acids
