# Supplementary material for: Microbiota and short chain fatty acid relationships underlie clinical heterogeneity and identify key microbial targets in irritable bowel syndrome (IBS)
Source: Sci Rep. 2025 Oct 9;15:35375. doi: 10.1038/s41598-025-19363-2 (PMC12511408; doi:10.1038/s41598-025-19363-2)

**Supplemental Figure 1:** β-Diversity Ordination of Samples from Health Control and Irritable Bowel Syndrome (IBS) Participants Including IBS with Constipation (IBS-C) and IBS with Diarrhea (IBS-D)


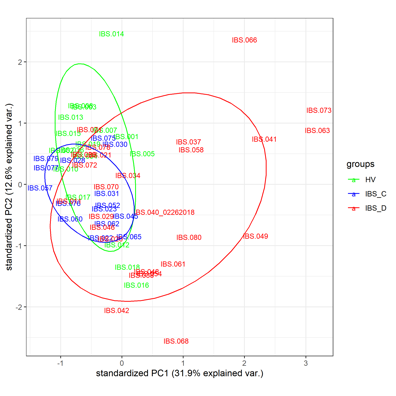

Supplement: Supplementary file 1 — Supplementary Material 1 [file 41598_2025_19363_MOESM1_ESM.docx]
