# Supplementary material for: Microbiota and short chain fatty acid relationships underlie clinical heterogeneity and identify key microbial targets in irritable bowel syndrome (IBS)
Source: Sci Rep. 2025 Oct 9;15:35375. doi: 10.1038/s41598-025-19363-2 (PMC12511408; doi:10.1038/s41598-025-19363-2)

**Supplemental Figure 2:** Partial Canonical Correspondence Analysis on Microbiome and Short Chain Fattty Acid Data, Conditioned on Transit Time, in Individual Clinical Groups


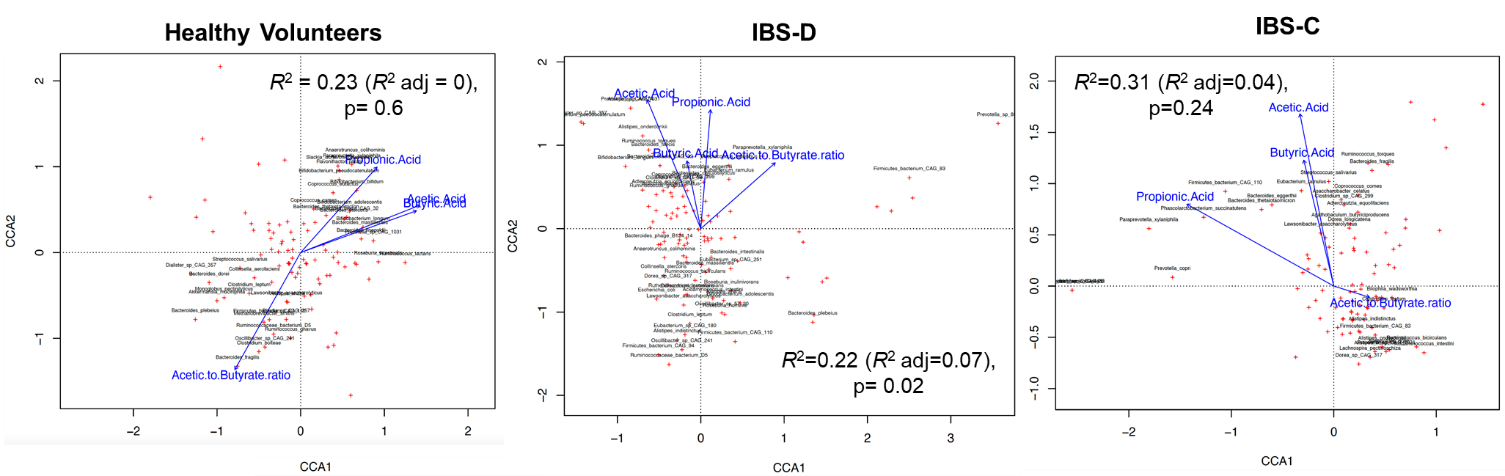

Supplement: Supplementary file 2 — Supplementary Material 2 [file 41598_2025_19363_MOESM2_ESM.docx]
