# Supplementary material for: Microbiota and short chain fatty acid relationships underlie clinical heterogeneity and identify key microbial targets in irritable bowel syndrome (IBS)
Source: Sci Rep. 2025 Oct 9;15:35375. doi: 10.1038/s41598-025-19363-2 (PMC12511408; doi:10.1038/s41598-025-19363-2)

**Supplemental Figure 3:** Bayesian Network Analysis of Microbial, Metabolite, and Transit Interactions in Healthy Volunteers (HV), IBS with diarrhea (IBS-D), and IBS with constipation (IBS-C)


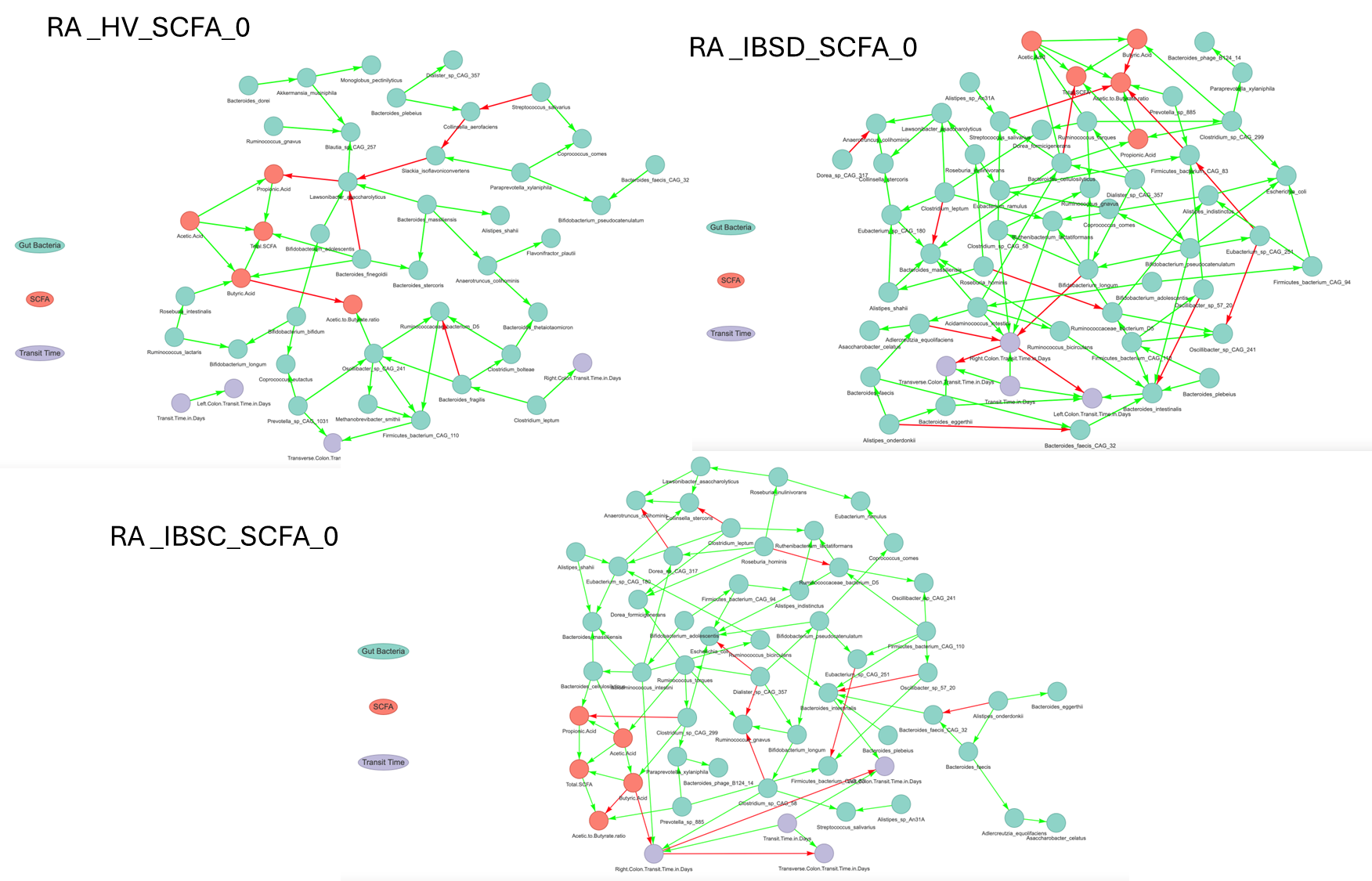

Supplement: Supplementary file 3 — Supplementary Material 3 [file 41598_2025_19363_MOESM3_ESM.docx]
