# Supplementary material for: Microbiota and short chain fatty acid relationships underlie clinical heterogeneity and identify key microbial targets in irritable bowel syndrome (IBS)
Source: Sci Rep. 2025 Oct 9;15:35375. doi: 10.1038/s41598-025-19363-2 (PMC12511408; doi:10.1038/s41598-025-19363-2)

**Supplemental Figure 4:** Non-metric multidimensional scaling (NMDS) representation of beta diversity in patients with (yes) and without (no) bile acid malabsorption (BAM) using taxonomic classification from MetaPhlAn.


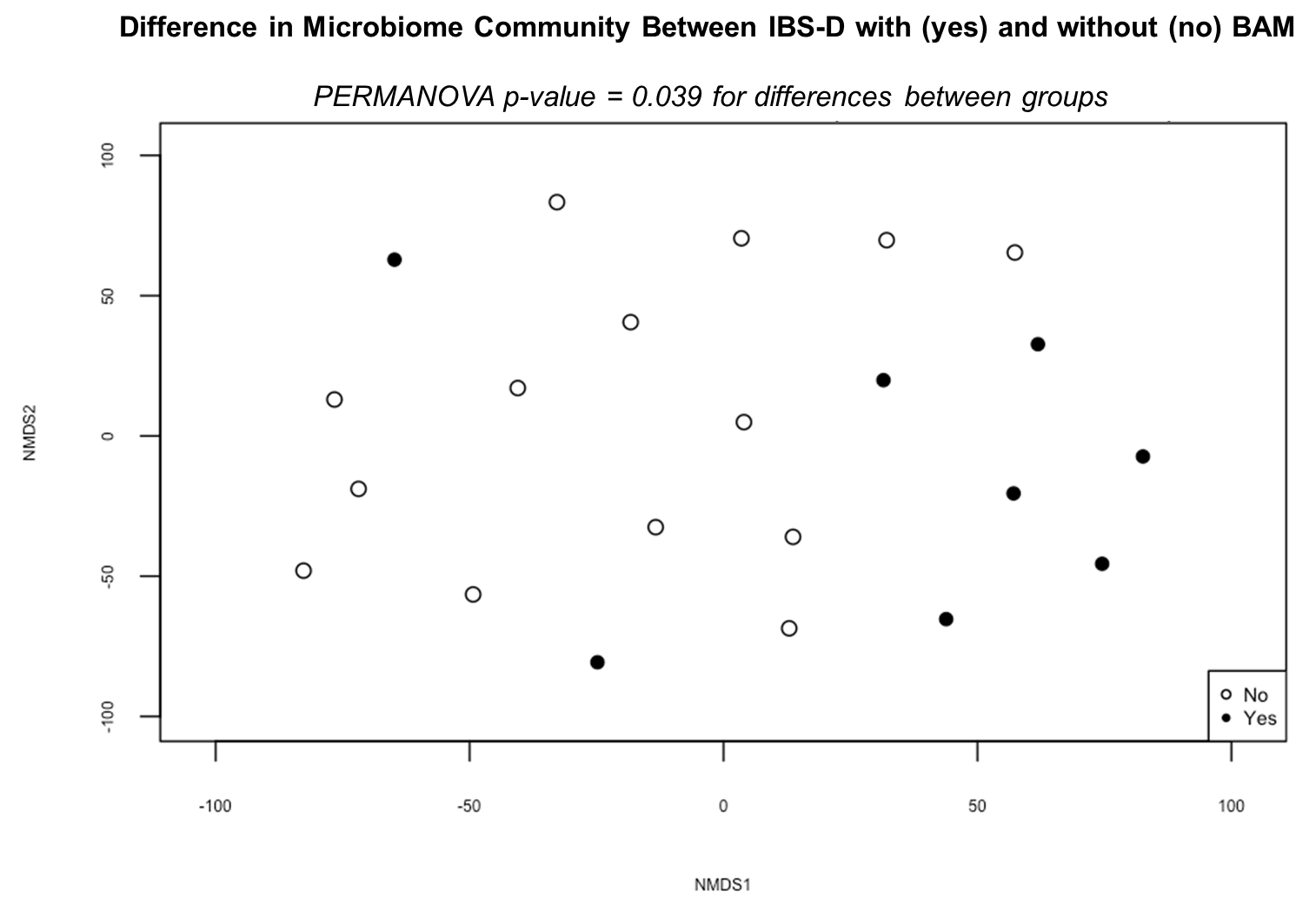

Supplement: Supplementary file 4 — Supplementary Material 4 [file 41598_2025_19363_MOESM4_ESM.docx]
